# Magnolol alleviates high-fat diet-induced steatosis, inflammation and fibrosis in obesity-associated liver injuries via MOGAT1-mediated lipid metabolism and NF-κB signaling

**DOI:** 10.3389/fphar.2026.1971993

**Published:** 2026-10-27

**Authors:** Ziqing Huang, Zixia Huang, Linfei Luo, Zhengqiang Wu, Zhili Wen, Zhihua Xiao

**Affiliations:** Department of Gastroenterology, The Second Affiliated Hospital, Jiangxi Medical College, Nanchang University, Nanchang, Jiangxi, China

**Keywords:** inflammation, liver, magnolol (PubChem CID: 72300), MOGAT1 gene, NF- kappa B, obesity, steatosis

## Abstract

**Background:**

Magnolol (MAG), a bioactive compound from Magnolia officinalis, possesses anti-inflammatory and lipid-lowering properties, but its role in obesity-associated liver injury and its molecular targets remain unknown.

**Methods:**

High-fat diet (HFD)-fed mice were treated with MAG (10 or 20 mg/kg) daily for 7 days. AML12 hepatocytes were challenged with PA with or without MAG pretreatment. Liver injury was evaluated by histology, serum biochemistry, qPCR and Western blot. RNA-seq and mechanistic studies were performed to identify MAG-regulated pathways and targets.

**Results:**

MAG significantly reduced hepatic lipid accumulation, serum transaminases, and collagen deposition in HFD-fed mice. RNA-seq identified monoacylglycerol O-acyltransferase 1 (MOGAT1) as the most significantly downregulated lipogenic gene by MAG. MOGAT1 expression correlated positively with liver triglyceride and injury markers. MAG also suppressed HFD-induced NF-κB activation (reduced P65 phosphorylation and nuclear translocation, lower IL-6, TNF-α, IL-1β). These findings were recapitulated in PA-treated AML12 cells. Critically, MOGAT1 overexpression largely reversed MAG-mediated suppression of inflammatory and fibrotic responses, demonstrating a causal role of MOGAT1 in MAG’s protective effects.

**Conclusion:**

MAG alleviates HFD-induced hepatic steatosis, inflammation and fibrogenic responses by suppressing MOGAT1-mediated triglyceride synthesis and subsequent NF-κB-driven inflammation. This study identifies MOGAT1 as a novel downstream effector and functional mediator of MAG, providing new mechanistic insight into its hepatoprotective action and supporting its therapeutic potential for obesity-associated liver injuries.

## 1 Introduction

The global prevalence of obesity-related conditions has emerged as a significant health and socio-economic challenge (Djeunankan et al., 2025). Lipid overload poses a substantial threat to liver health by promoting chronic inflammation and fibrosis, which contribute to non-alcoholic fatty liver disease (NAFLD) and its advanced form non-alcoholic steatohepatitis (NASH) (London and George, 2007; Afonso et al., 2024). Despite the growing medical needs, modern pharmaceuticals have yet to provide effective treatments for NASH or NAFLD, presenting an unprecedented challenge for researchers (Cobbina and Akhlaghi, 2017).

The mechanisms underlying obesity-associated hepatic inflammation and fibrosis remain unclear. The activation of Nuclear factor kappa-B (NF-κB) signaling plays a crucial role in initiating the pro-inflammatory response by producing inflammatory mediators such as tumor necrosis factor-α (TNF-α), interleukin-6 (IL-6), and interleukin-1β (IL-1β). Additionally, NF-κB signaling is involved in the progression of hepatic fibrosis through interactions with hepatocytes, Kupffer cells, and stellate cells (Catrysse et al., 2016). Furthermore, excessive reactive oxygen species (ROS) generated by lipotoxicity and inflammation exacerbate liver damage, leading to mitochondrial dysfunction and hepatocyte loss. This deterioration precipitates inflammation and fibrogenesis, ultimately contributing to end-stage liver fibrosis and cirrhosis (Xu et al., 2021). Lipid accumulation itself is a key driver of hepatic inflammation, and enzymes involved in triglyceride synthesis have emerged as potential therapeutic targets. Among these, monoacylglycerol O-acyltransferase 1 (MOGAT1) catalyzes the conversion of monoacylglycerol to diacylglycerol, a rate-limiting step in triglyceride biosynthesis (Wei et al., 2025). Hepatic MOGAT1 expression is elevated in obesity and NAFLD, but its regulation by natural products remains largely unexplored (Won et al., 2026; Hall et al., 2012).

Magnolol (5,5′-diallyl-2,2′-dihydroxybiphenyl, MAG) is a bioactive biphenolic compound isolated from the bark and cones of Magnolia officinalis, a plant widely used in traditional Chinese medicine. MAG has garnered extensive attention for its diverse pharmacological activities, including anti-inflammatory, antioxidant, anti-tumor, and neuroprotective effects (Wang et al., 2025; Dai et al., 2024). Numerous studies have demonstrated that MAG can suppress the activation of NF-κB and modulate the NRF2 antioxidant pathway, as well as inhibit the NLRP3 inflammasome, thereby ameliorating inflammatory conditions in various tissues (Lv et al., 2026; Gwon et al., 2026; Tao et al., 2021). Moreover, MAG has been reported to activate AMPK and regulate lipid metabolism in adipose tissue and skeletal muscle (Tian et al., 2018; Ge et al., 2020; Chen et al., 2015). However, whether MAG can alleviate obesity-associated hepatic steatosis and fibrosis, and whether it acts through modulating MOGAT1, remains to be investigated.

In this study, we examined the hepatoprotective effects of MAG on high-fat diet (HFD)-induced liver steatosis, inflammation and fibrosis. Through unbiased RNA-seq analysis, we identified MOGAT1 as a novel downstream functional mediator of MAG. Our findings suggest that MAG alleviates NAFLD by suppressing MOGAT1-mediated triglyceride synthesis and subsequent NF-κB-driven inflammation, revealing a previously unrecognized mechanism of action for this natural compound.

## 2 Materials and methods

### 2.1 Animals

8-week-old male C57BL/6J mice (n = 30) specific pathogen-free (SPF) weighing 20–25 g were purchased from VitaRiver Co. (Beijing, China). All experimental procedures were approved by the Nanchang University’s Institutional Animal Care and Use Committee and conducted in accordance with the recommendations of the National Institutes of Health Guide for Care and Use of Laboratory Animals and adhered to the ARRIVE guidelines (Approval Document No. NCULAE-20250516001). The mice were housed in the Animal Experiment Center of Nanchang university.

Mice were randomly allocated to 5 groups (n = 6 per group): (1) control (vehicle, chow diet); (2) MAG alone (20 mg/kg, chow diet); (3) HFD + vehicle; (4) HFD + MAG (10 mg/kg); (5) HFD + MAG (20 mg/kg). MAG was dissolved in corn oil and administered daily via intraperitoneal injection. Intraperitoneal injection was chosen in this proof-of-concept study to ensure precise and reproducible dosing, as oral gavage of MAG in an oil vehicle can result in variable absorption due to its lipophilic nature and extensive first-pass metabolism. While this route differs from the traditional oral use of Magnolia officinalis, it allows unambiguous assessment of the pharmacological effects of MAG at controlled systemic exposure levels. The control group and MAG alone group were fed a chow diet, while the remaining groups were fed a HFD containing 60 kcal% fat, 20 kcal% protein and 20 kcal% carbohydrates (cat. #MD12033, MediScience, China) for 16 weeks, followed by 7-day treatment with MAG or vehicle.

### 2.2 MAG preparation

MAG (purity > 98%) was purchased from Solarbio (Beijing, China). According to the manufacturer’s certificate, the compound was isolated from the bark of Magnolia officinalis by ethanol extraction, silica gel column chromatography, and recrystallization, with purity confirmed by High Performance Liquid Chromatography (HPLC).

### 2.3 Cell culture

The mouse liver cell AML12 was obtained from China Center for Type Culture Collection (CCTCC) and cultured in Dulbecco’s Modified Eagle’s Medium (DMEM)-F12 medium (Gibco, United States) supplemented with 10% Fetal bovine serum (FBS; Gibco), 100 U/mL penicillin/streptomycin (Beyotime Biotechnology, China), 1:500 insulin-transferrin-selenium (Corning, United States), 40 ng/mL dexamethasone (Sigma-Aldrich, United States) at 37 °C in a 5% CO_2_ atmosphere. All reagents used for cell culture and animal experiments, including magnolol, were confirmed to be endotoxin-free (<0.1 EU/mL) as determined by the limulus amebocyte lysate assay (GenScript, China). Cells were treated with 200 µM of sodium palmitate (PA; Sigma-Aldrich, United States) for *in vitro* studies. MAG was added at a concentration of 5 or 10 μmol/L 1 h prior to PA stimulation (24 h).

For MOGAT1 overexpression experiments, AML12 cells were transfected with pcDNA3.1-MOGAT1 overexpression plasmid or empty vector (GenePharma, China) using Lipofectamine 3000 (Invitrogen) according to the manufacturer’s protocol. All cell experiments were performed with at least three independent biological replicates, each consisting of three technical replicates.

### 2.4 Histological assessment

Liver tissues were fixed with 4% paraformaldehyde, embedded in paraffin and sliced into 4 μm sections. Hematoxylin and eosin (H&E) staining was performed using H&E staining kit (Beyotime Biotechnology). Sirius red and Masson’s trichrome staining were conducted using Sirius red staining kit and Masson’s trichrome staining kit (Solarbio, China). Frozen sections were used for Oil red O staining using Oil red O staining kit (Beyotime Biotechnology). All procedures were in accordance with the guidelines of the manufacturers’ instructions. For quantification of Oil Red O, Sirius red, and Masson’s trichrome staining, 5 random fields per animal were examined at ×200 magnification. All quantitative analyses were performed independently by two investigators blinded to the treatment groups using ImageJ software (NIH, United States), and the mean value of the 5 fields was taken as the representative value for each animal.

### 2.5 Liver enzymes and lipid measurement

Serum alanine aminotransferase (ALT) and aspartate aminotransferase (AST) levels were detected using ALT Activity Assay Kit and AST Activity Assay Kit (Elabscience, China). Serum concentrations of low-density lipoprotein (LDL) and triglyceride (TG) were determined using the Mouse LDL ELISA Kit and TG Content Assay Kit (Sangon Biotech, China). Serum total cholesterol (TCH) concentration was evaluated using Total Cholesterol Assay Kit (Yeasen Biotechnology, China).

### 2.6 RNA sequencing (RNA-seq)

Total RNA was extracted from liver tissues using TRIzol reagent (Invitrogen, CA, United States) following the manufacturer’s protocol. Samples were frozen in liquid nitrogen, stored in dry ice and sent to OmicShare (China) for further processing. Sequencing was conducted using an Illumina NovaseqTM 6000 with three biological replicates per group. Raw reads were aligned to the mouse reference genome GRCm38 (mm10) using HISAT2. Differential expression analysis was performed using DESeq2 with thresholds of |log2 fold change| > 1 and adjusted P value < 0.05 (Benjamini–Hochberg correction). Functional enrichment analysis was performed using the clusterProfiler package in R software with Benjamini–Hochberg correction for multiple testing.

### 2.7 Real-time polymerase chain reaction (PCR)

RNA was extracted from tissues or cells using TRIzol reagent (Invitrogen). Synthesis of cDNA was achieved by using M-MLV reverse transcriptase (Promega, United States). The mRNA levels of genes were evaluated using TB Green® Premix Ex Taq™ II (TAKARA, JAPAN) in StepOnePlus Real-Time PCR System (Applied Biosystems, United States). *Gapdh* served as the internal control. The primers used in this study were obtained from Sangon Biotech (China) and were listed as follows.

**Table:** 

Gene	Forward (5′-3′)	Reverse (5′-3′)
*Srebp1*	GCA​GCC​ACC​ATC​TAG​CCT​G	CAG​CAG​TGA​GTC​TGC​CTT​GAT
*Acaca*	GAT​GAA​CCA​TCT​CCG​TTG​GC	GAC​CCA​ATT​ATG​AAT​CGG​GAG​TG
*Fasn*	TAT​CAA​GGA​GGC​CCA​TTT​TGC	TGT​TTC​CAC​TTC​TAA​ACC​ATG​CT
*Col1a1*	AAT​GGT​GCT​CCT​GGT​ATT​GC	GGT​CCT​CGT​TTT​CCT​TCT​T
*Tgfb1*	CTC​CCG​TGG​CTT​CTA​GTG​C	GCC​TTA​GTT​TGG​ACA​GGA​TCT​G
*Acta2*	GTC​CCA​GAC​ATC​AGG​GAG​TAA	TCG​GAT​ACT​TCA​GCG​TCA​GGA
*Il6*	TAG​TCC​TTC​CTA​CCC​CAA​TTT​CC	TTG​GTC​CTT​AGC​CAC​TCC​TTC
*Tnf*	CAG​GGG​CCA​CCA​CGC​TCT​TC	TTT​GTG​AGT​GTG​AGG​GTC​TGG
*Il1b*	ACT​CCT​TAG​TCC​TCG​GCC​A	CCA​TCA​GAG​GCA​AGG​AGG​AA
*Mogat1*	CTG​CTA​CCG​CTT​CCT​CAG​AG	TCC​AGT​TGA​TGC​CAT​TGA​GG
*Gapdh*	GTG​GCA​AAG​TGG​AGA​TTG​TTG	CGT​TGA​ATT​TGC​CGT​GAG​TG

### 2.8 Western blot analysis

Proteins prepared from tissues or cells were separated on 10% sodium dodecyl sulfate (SDS)-polyacrylamide gel electrophoresis and transferred to a polyvinylidene difluoride (PVDF) membrane (Bio-Rad, United States). Membranes were blocked in 5% nonfat dry milk for 1 h at room temperature and incubated with the primary antibodies at 4 °C overnight. Then, membranes were washed by Tris-buffered saline with 0.1% Tween 20 (TBS-T) and incubated with Horseradish peroxidase (HRP)-conjugated secondary antibodies (Proteintech, China) in 5% non-fat milk in TBS-T buffer for 1 h at room temperature. Detection was carried out using an image scanner (Quantity One System; Bio-Rad, United States). Primary antibodies used included: anti-phospho-P65 (Ser536; 1:500; Proteintech, China), anti-P65 (1:1,000; Proteintech, China), anti-IκBα (1:500; Proteintech, China), anti-GAPDH (1:2,000; HUABIO, Hangzhou, China), anti-Histone H3 (1:1,000; Cell Signaling Technology, United States), anti-IL6 (1:800; Proteintech, China), anti-TNF-α (1:1,000; Proteintech, China), anti-IL-1β (1:1,000; Proteintech, China), anti-COL1 (1:1,000; Proteintech, China), anti-TGF-β1 (1:500; Proteintech, China), anti-ACTA2 (1:1,000; Proteintech, China), and anti-MOGAT1 (1:500; Santa Cruz Biotechnology, United States).

### 2.9 Nuclear/cytoplasmic fractions

The nuclear and cytoplasmic fractions of tissues or cells were separated using the Nuclear and Cytoplasmic Protein Extraction Kit (Beyotime Biotechnology) according to the guidelines. A mix of protease inhibitors (Boehringer, Germany) was added to the fractions to inhibit protein degradation.

### 2.10 Immunofluorescence assay

Sections from mouse liver tissues were deparaffinized, followed by antigen retrieval and blocking in QuickBlock™ Blocking Buffer for Immunol Staining (Beyotime Biotechnology) for 1 h at room temperature. Then, sections were incubated with rabbit anti-P65 antibody (1:100 dilution, 10745-1-AP, Proteintech) at 4 °C overnight. Washed by phosphate-buffered saline (PBS), the sections were incubated with Goat anti-Rabbit IgG (H + L) Cross-Adsorbed Secondary Antibody, Alexa Fluor™ 546 (Thermo Fisher, United States) for 1 h at room temperature. Nucleus was stained by 4′,6-diamidino-2-phenylindole (DAPI). For *in vitro* study, cells were fixed with 4% paraformaldehyde, followed by 0.2% (V/V) Triton X-100 permeabilization for 5 min and blocking before antibody incubation. Images were captured under a fluorescent microscope (Olympus, Japan). For quantitative analysis, 5 random fields per sample were captured, and fluorescence intensity was measured by two independent investigators blinded to the experimental groups using ImageJ software.

### 2.11 Dihydroethidium (DHE) staining

DHE (Beyotime Biotechnology) was dissolved in PBS (20 mM) before incubation with cells at a dilution of 2 μM. Images were captured under a fluorescent microscope (Olympus, Japan) and analysis of fluorescence intensity was conducted using ImageJ software (NIH, United States).

### 2.12 Data availability

The RNA-seq data presented in this study are available from the corresponding author upon reasonable request.

### 2.13 Statistical analysis

All data were expressed as the mean ± standard deviation (SD). Statistical analyses were performed with GraphPad Prism 9 (GraphPad Software, United States). Normality of distribution was assessed with Shapiro–Wilk test. Two groups with normal distributions were compared using two-sided unpaired Student’s *t*-tests. Comparisons of multiple groups were carried out by one-way analysis of variance (ANOVA) followed by Bonferroni or Dunnett’s *post hoc* analysis. Correlation analyses between MOGAT1 expression and the indicated biochemical parameters (hepatic TG, serum ALT, and inflammatory cytokine mRNA level) were performed using Spearman’s rank correlation coefficient. A two-tailed P value < 0.05 was considered as significant.

## 3 Results

### 3.1 MAG alleviates HFD-induced hepatic steatosis and liver dysfunction

To evaluate the therapeutic potential of MAG in NAFLD, we first assessed basic physiological and biochemical parameters in HFD-fed mice following MAG treatment. Overall body weight was significantly higher in HFD-fed groups compared to chow-fed controls, but was not affected by MAG treatment (Figure 1A). However, the liver-to-body weight ratio was markedly reduced by MAG administration in a dose-dependent manner (Figure 1B). Serum ALT and AST levels, which reflect hepatocellular injury, were significantly elevated in HFD-fed mice, and both were dose-dependently decreased by MAG treatment (Figure 1C). Serum levels of TG, TCH, and LDL were consistently decreased by MAG, suggesting its regulatory role in systemic lipid metabolism (Figure 1D). H&E and Oil Red O staining revealed marked lipid droplet accumulation in HFD-fed mouse livers, which was significantly attenuated by MAG treatment (Figure 1E). Consistent with these findings, the transcriptional levels of lipogenesis-associated genes, including Srebp1, Acaca, and Fasn, were suppressed by MAG in response to HFD stimulation (Figure 1F). Collectively, these findings demonstrate that MAG exerts hepatoprotective effects against HFD-induced hepatic steatosis and liver dysfunction.

**FIGURE 1:**
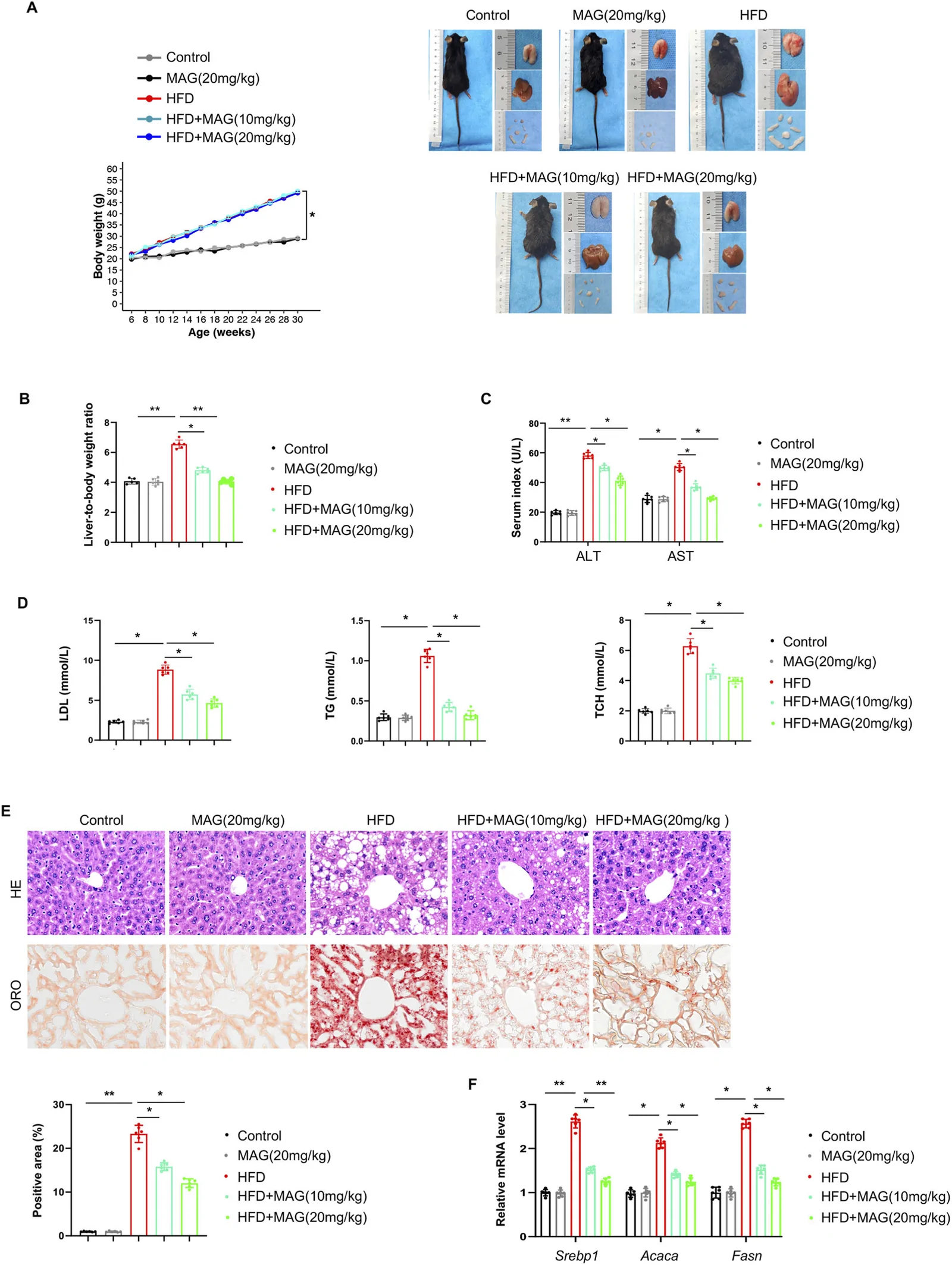
MAG alleviates HFD-induced hepatic steatosis and liver dysfunction. **(A)** Body weight (left) and representative images of body and organs (right). **(B)** Liver-to-body weight ratio. **(C)** Serum ALT and AST levels. **(D)** Serum lipids (TG, TCH, LDL) levels. **(E)** Representative H&E and Oil Red O staining of liver sections (left) with quantification of Oil Red O-positive area (right). Scale bar = 20 μm. **(F)** qPCR analysis of lipogenic genes (*Srebp1, Acaca, Fasn*) in liver tissues. Data are mean ± SD. *P < 0.05, **P < 0.01.

### 3.2 MAG attenuates HFD-induced hepatic fibrogenic responses

Liver fibrosis is a critical pathological manifestation of obesity-induced liver injury. We therefore examined the effects of MAG on hepatic fibrosis. Sirius red and Masson’s trichrome staining revealed significant collagen deposition in the livers of HFD-fed mice, which was markedly reduced by MAG treatment (Figure 2A). Consistently, both mRNA and protein levels of fibrotic markers, including collagen type I (COL1), transforming growth factor-β1 (TGF-β1), and actin alpha 2, smooth muscle (ACTA2), were significantly elevated in HFD-fed mice and normalized by MAG treatment (Figures 2B,C). These data indicate that MAG effectively attenuates active fibrogenesis in HFD-fed mice, as reflected by reduced collagen deposition and downregulation of fibrotic marker expression.

**FIGURE 2:**
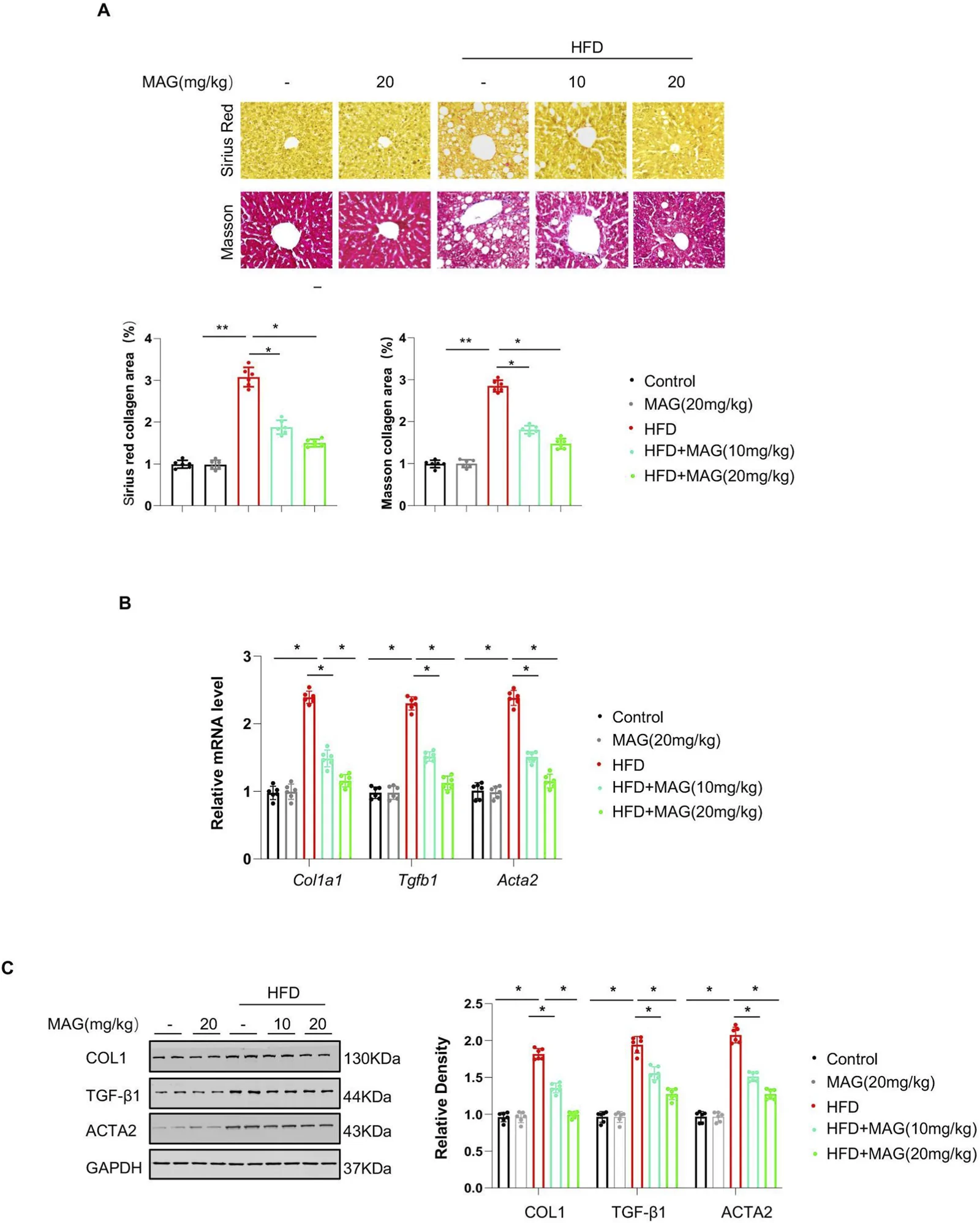
MAG reduces HFD-induced liver fibrosis. **(A)** Representative Sirius red and Masson’s trichrome staining of liver sections (left) with quantification of collagen-positive area (right). Scale bar = 20 μm. **(B)** qPCR analysis of fibrotic genes (*Col1a1, Tgfb1, Acta2*). **(C)** Western blot analysis of fibrotic proteins (COL1, TGF-β1, ACTA2). Data are mean ± SD. *P < 0.05, **P < 0.01.

### 3.3 RNA-seq identifies MOGAT1 as a novel downstream effector of MAG

To elucidate the molecular mechanisms underlying the hepatoprotective effects of MAG, we performed RNA-seq analysis on liver tissues from control, HFD, and HFD + MAG (20 mg/kg) groups. Volcano plot analysis showed that 476 genes were upregulated and 380 genes were downregulated by HFD compared to chow-fed controls. Upon HFD challenge, 372 genes were upregulated and 194 genes were downregulated by MAG treatment (Figure 3A). Importantly, 69 genes that were induced by HFD were significantly reversed by MAG (Figure 3B; Supplementary Table S1). KEGG pathway analysis of these MAG-reversed genes revealed enrichment in pathways concerning inflammatory signaling (Figure 3C).

**FIGURE 3:**
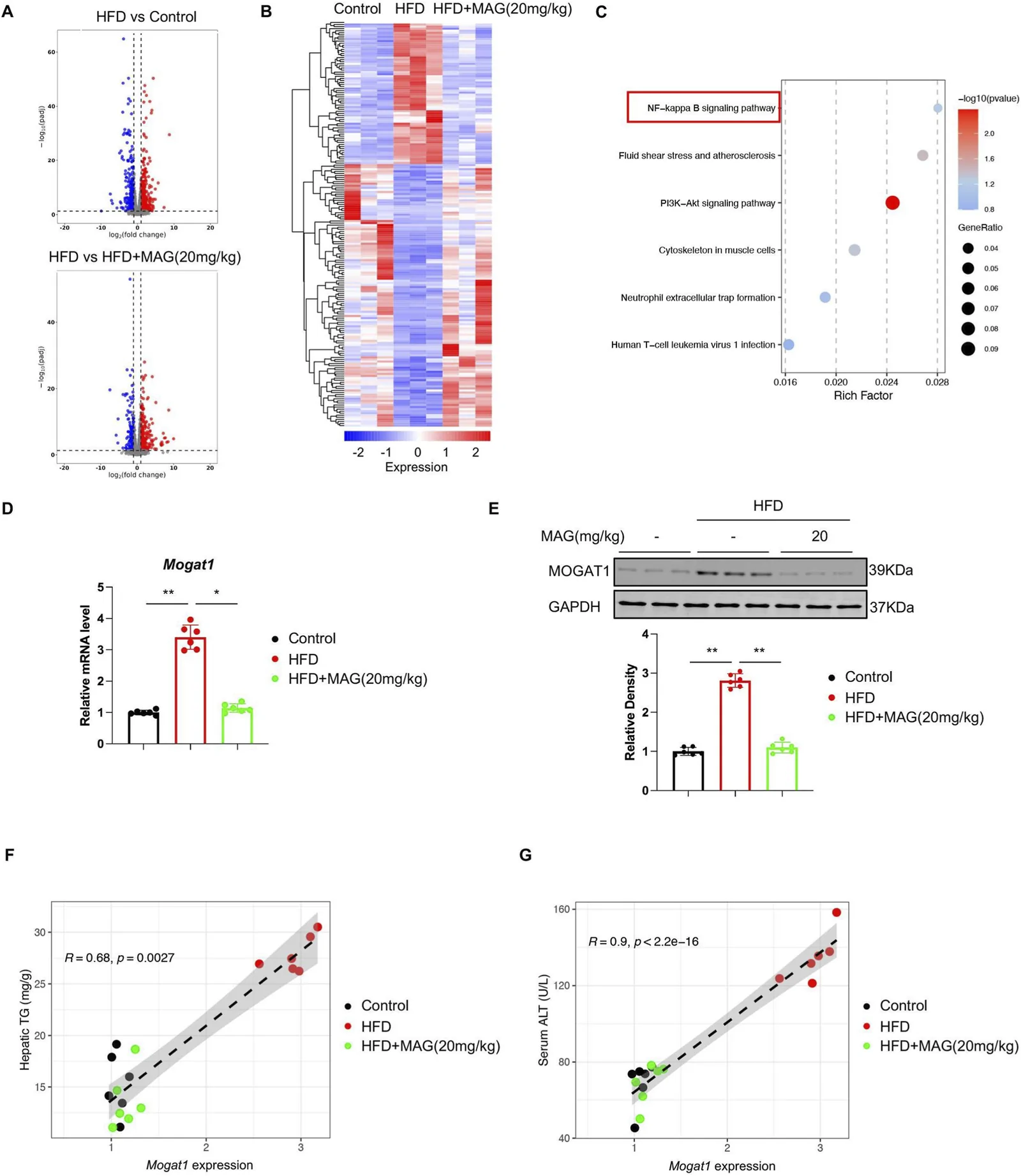
RNA-seq identifies MOGAT1 as a novel downstream effector of MAG. **(A)** Volcano plots of DEGs between HFD vs. control (left) and HFD + MAG vs. HFD (right). **(B)** Heatmap showing genes significantly altered by MAG upon HFD challenge. **(C)** KEGG pathway enrichment of MAG-reversed genes. **(D)** mRNA expression of *Mogat1* in liver tissues. **(E)** Western blot analysis of MOGAT1 protein in liver tissues. **(F,G)** Correlation of *Mogat1* expression with hepatic TG content **(F)** and serum ALT **(G)** across all individual samples (black, Control; red, HFD; green, HFD + MAG). Data are mean ± SD. *P < 0.05, **P < 0.01.

Among the 69 MAG-reversed genes, we focused on MOGAT1 because it encodes a rate-limiting enzyme in triglyceride synthesis and was the most significantly downregulated lipid-metabolizing enzyme whose hepatic function in NAFLD remains incompletely understood (log2FC = −2.521, adjusted P < 0.001; Supplementary Table S2) (Won et al., 2026; Liss et al., 2021). MAG treatment significantly suppressed HFD-induced MOGAT1 expression at both mRNA and protein levels (Figures 3D,E). Importantly, hepatic MOGAT1 expression correlated positively with liver triglyceride (TG) content and serum ALT levels across all individual samples, suggesting a functional link between MOGAT1 and hepatic steatosis and injury (Figures 3F,G). These findings identify MOGAT1 as a previously unrecognized downstream effector of MAG in the liver.

### 3.4 MAG inhibits HFD-induced NF-κB signaling in the liver

Given the established role of NF-κB in hepatic inflammation, we examined whether MAG affects this pathway. Western blot analysis revealed that HFD feeding significantly increased the phosphorylation of P65 (p-P65) and reduced IκBα protein levels, indicating activation of the canonical NF-κB pathway. MAG treatment reversed these effects (Figure 4A). Nuclear-cytoplasmic fractionation and immunofluorescence staining further confirmed that MAG inhibited HFD-induced nuclear translocation of P65 (Figures 4B,C). Consistently, mRNA and protein levels of NF-κB-dependent pro-inflammatory cytokines (TNF-α, IL-6, IL-1β) were significantly elevated in HFD-fed mice and reduced by MAG treatment (Figures 4D,E). Of note, MOGAT1 expression showed a significant positive correlation with TNF-α mRNA level across samples (Figure 4F), suggesting that MOGAT1-mediated lipid accumulation may drive NF-κB activation.

**FIGURE 4:**
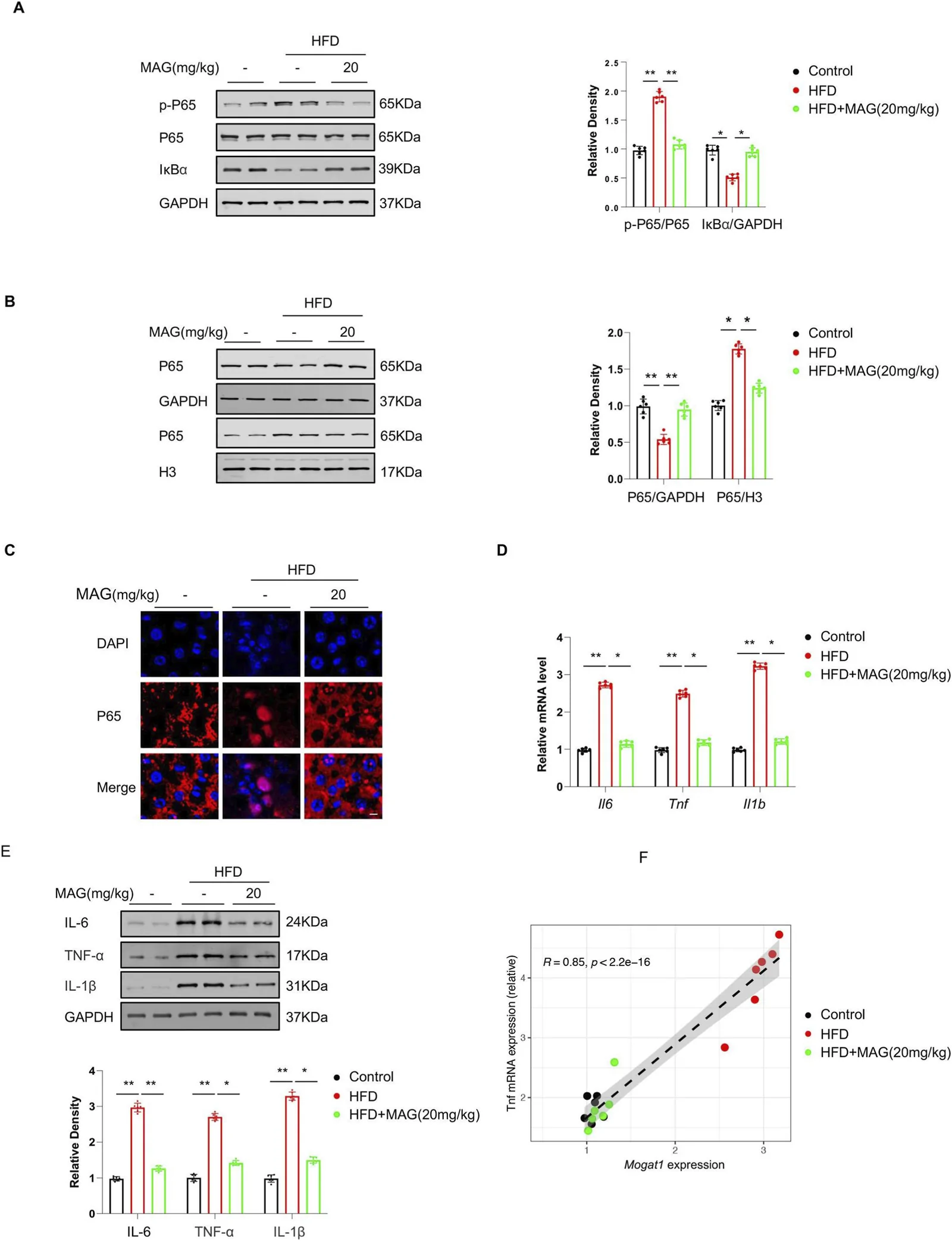
MAG inhibits HFD-induced NF-κB signaling in the liver. **(A)** Western blot analysis of p-P65, P65, and IκBα in liver tissues. **(B)** Nuclear-cytoplasmic fractionation showing nuclear P65 translocation. **(C)** Immunofluorescence staining of P65 (red) and DAPI (blue) in liver sections. Scale bar = 5 μm. **(D)** qPCR analysis of pro-inflammatory cytokines (*Il6, Tnf, Il1b*). **(E)** Western blot analysis of IL-6, TNF-α, and IL-1β. **(F)** Correlation between *Mogat1* and *Tnf* mRNA expression (black, Control; red, HFD; green, HFD + MAG). Data are mean ± SD. *P < 0.05, **P < 0.01.

### 3.5 MAG relieves PA-induced inflammation and fibrogenic responses in AML12 cells

We next validated the hepatoprotective effects of MAG *in vitro* using PA-challenged AML12 hepatocytes. PA treatment significantly upregulated MOGAT1 expression at both mRNA and protein levels, while MAG pretreatment reversed this upregulation in a concentration-dependent manner (Figures 5A,B). To determine whether MAG’s anti-inflammatory effect and attenuation of fibrogenic responses are causally dependent on MOGAT1 suppression, we performed a rescue experiment by overexpressing MOGAT1 in AML12 cells. Overexpression of MOGAT1 was confirmed at both mRNA and protein levels (Figures 5C,D). As expected, PA stimulation induced P65 phosphorylation and nuclear translocation, which were markedly inhibited by MAG treatment in vector-transfected cells. Notably, MOGAT1 overexpression largely reversed MAG-mediated suppression of P65 phosphorylation and nuclear translocation (Figures 5E–G). Consistently, MAG-induced downregulation of pro-inflammatory cytokines (IL-6, TNF-α, IL-1β) and fibrotic markers (COL1, TGF-β1, ACTA2) were significantly abrogated by MOGAT1 overexpression at both mRNA and protein levels (Figures 5H–K). Furthermore, MAG-mediated reduction of ROS production was also attenuated by MOGAT1 overexpression (Figure 5L). Collectively, these data confirm that MAG directly acts on hepatocytes to alleviate PA-induced inflammation, fibrogenic gene expression, and oxidative stress, and that MOGAT1 suppression is a critical mediator of these protective effects.

**FIGURE 5:**
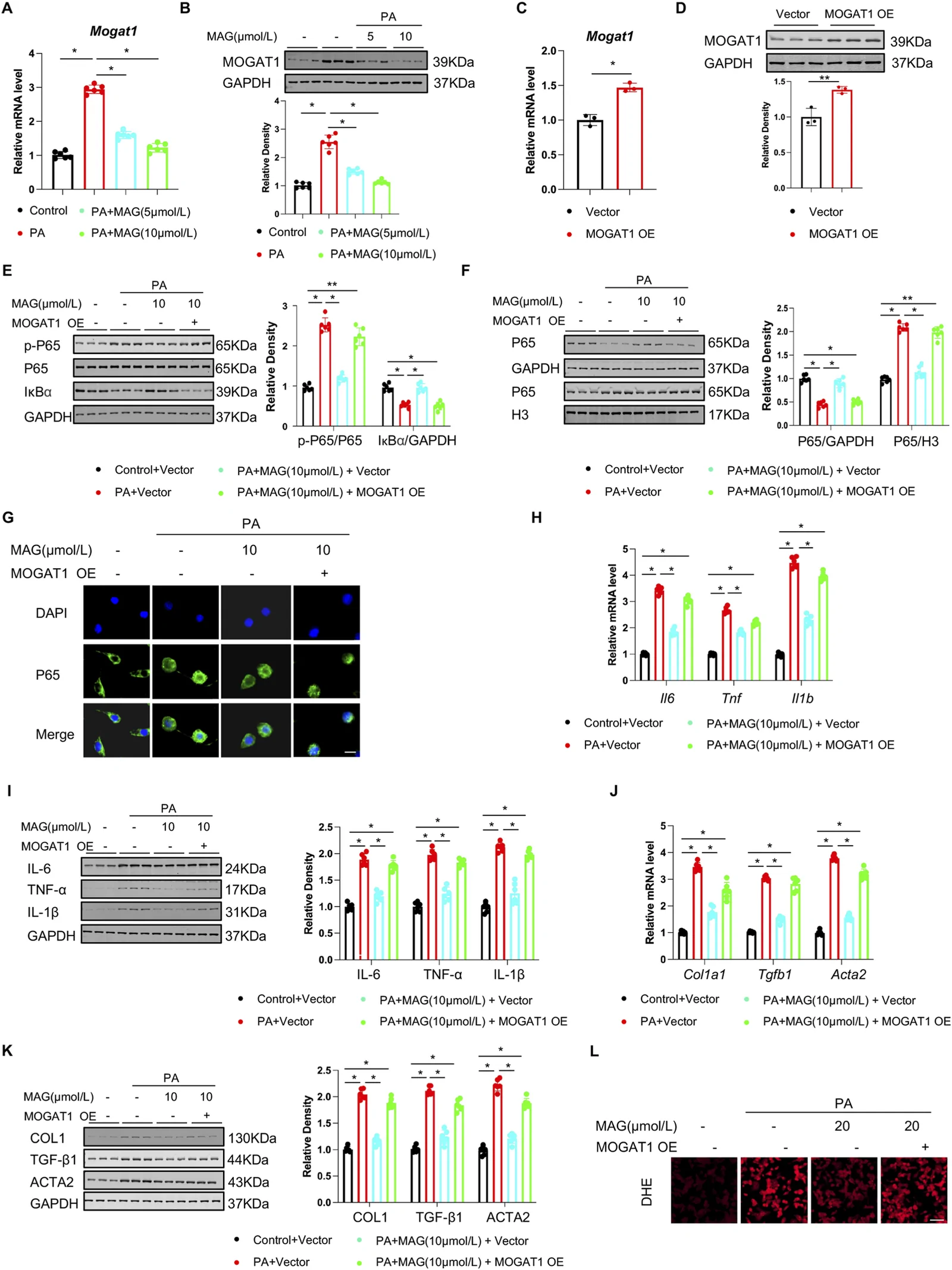
MAG alleviates PA-induced inflammation and fibrogenic responses in AML12 cells through MOGAT1-dependent mechanism. AML12 cells were transfected with MOGAT1-overexpressing plasmid (MOGAT1 OE) or empty vector (Vector) for 24 h, followed by MAG pretreatment and then PA stimulation. **(A,B)** MAG suppressed PA-induced MOGAT1 mRNA **(A)** and protein **(B)** expression in a concentration-dependent manner. **(C,D)** Overexpression efficiency of MOGAT1 was confirmed by qPCR **(C)** and Western blot **(D)**. **(E)** Western blot analysis of phosphorylated P65 (p-P65), total P65, and IκBα in cells treated as indicated. **(F)** Nuclear-cytoplasmic fractionation showing P65 nuclear translocation. GAPDH, cytoplasmic control; H3, nuclear control. **(G)** Immunofluorescence staining of P65 (green) and DAPI (blue) showing subcellular localization of P65. Scale bar = 10 μm. **(H)** qPCR analysis of pro-inflammatory cytokines (*Il6, Tnf, Il1b*). **(I)** Western blot analysis of IL-6, TNF-α, and IL-1β proteins. **(J)** qPCR analysis of fibrotic genes (*Col1a1, Tgfb1, Acta2*). **(K)** Western blot analysis of COL1, TGF-β1, and ACTA2 proteins. **(L)** Representative DHE staining for ROS (left) and quantification of fluorescence intensity (right). Scale bar = 50 μm. Data are expressed as mean ± SD. *P < 0.05, **P < 0.01.

## 4 Discussion

Obesity is closely linked to inflammation and fibrosis during the development and progression of obesity-associated conditions, such as NAFLD. In this study, we, for the first time, demonstrate that MAG exerts potent hepatoprotective effects against HFD-induced hepatic steatosis, inflammation, and fibrosis in mice, and we identify MOGAT1 as a novel functional mediator of MAG’s effects. The causal role of Mogat1 in mediating MAG’s hepatoprotective effects was further substantiated by our rescue experiments in AML12 cells. Overexpression of MOGAT1 significantly reversed MAG-mediated suppression of NF-κB activation, inflammatory cytokine production, fibrotic gene expression, and ROS generation. These findings provide direct evidence that MOGAT1 is a functional downstream effector of MAG, rather than merely a correlated transcriptional change. To our knowledge, this is the first study to establish a causal link between MAG, MOGAT1 suppression, and resolution of hepatic inflammation and fibrosis.

Unhealthy lifestyles, particularly HFD consumption, contribute to obesity and facilitate a range of liver abnormalities, including lipid metabolism dysfunction, NASH, and NAFLD. Excessive inflammatory responses and fibrosis are key events in the progression of various liver diseases. For instance, TNF-α, IL-6, and IL-1β are classical pro-inflammatory factors in NF-κB signaling that are overproduced during NAFLD (He et al., 2026). Inflammation-induced collagen production and deposition are essential for the development of hepatic fibrosis (Wynn and Ramalingam, 2012). Additionally, lipid overload impairs redox homeostasis by promoting ROS generation, which exacerbates oxidative stress and activates pro-inflammatory signals (Li et al., 2020). Our findings demonstrate that MAG simultaneously addresses lipid accumulation and inflammation, making it a promising compound that concurrently addresses lipid accumulation and inflammation, with beneficial effects on fibrogenesis.

The most novel finding of this study is the identification of MOGAT1 as a key downstream effector of MAG. MOGAT1 catalyzes the conversion of monoacylglycerol to diacylglycerol, a rate-limiting step in triglyceride synthesis (Wei et al., 2025; Lyu et al., 2021). Hepatic MOGAT1 expression is elevated in obesity and insulin-resistant states, and its overexpression promotes hepatic steatosis (Ramon-Krauel et al., 2018). However, the regulation of MOGAT1 by natural products has remained largely unexplored. Our RNA-seq analysis revealed that MAG significantly suppresses HFD-induced MOGAT1 expression, and the reduction in hepatic MOGAT1 levels correlates strongly with improved steatosis and reduced liver injury. The magnitude of MOGAT1 transcriptional suppression was modest compared with some lipid droplet-associated genes; however, as the only significantly downregulated lipid-metabolizing enzyme among the MAG-reversed genes, and given its rate-limiting role in triglyceride synthesis, MOGAT1 represents a functionally decisive node. This is directly supported by our rescue experiments, in which MOGAT1 overexpression abolished MAG’s protective effects. Therefore, this is the first report linking MAG to MOGAT1 inhibition, providing a new molecular basis for its lipid-lowering effects.

Beyond its effects on MOGAT1, MAG also inhibited NF-κB signaling in HFD-fed mice and PA-treated hepatocytes. This is consistent with previous reports demonstrating the anti-inflammatory properties of MAG in other disease models (Liao et al., 2026; Yang et al., 2026). Importantly, we observed a significant positive correlation between MOGAT1 expression and inflammatory cytokine level, suggesting that MOGAT1-mediated lipid accumulation may serve as an upstream trigger for NF-κB activation. This is biologically plausible, as intracellular lipid overload can activate inflammatory signaling through multiple mechanisms, including ER stress, ROS production, and Toll-like receptor activation (Vesga-Jimenez et al., 2022; Zhou et al., 2020). Thus, we propose a mechanistic model in which MAG suppresses MOGAT1 to reduce lipid synthesis, thereby removing the lipotoxic stimulus that drives NF-κB-dependent inflammation. This dual action–directly inhibiting lipid synthesis and indirectly suppressing inflammation–may explain the robust hepatoprotective effects of MAG observed in our study. However, we acknowledge that other MAG-regulated genes—such as Scd1 and Cpt1a—may also contribute to the observed hepatoprotective phenotype. The overall benefit of MAG likely involves polygenic modulation rather than a single-gene effect, and future studies should systematically evaluate the combinatorial contributions of these genes.

Our findings have important translational implications. MAG is a naturally occurring compound with a well-characterized safety profile and has been used in traditional medicine for centuries. The doses used in this study are within the range reported in previous preclinical studies and are pharmacologically achievable (Tuli et al., 2026). However, whether oral administration of MAG achieves sufficient hepatic exposure to reproduce the observed hepatoprotective effects remains to be determined. Future pharmacokinetic and pharmacodynamic studies using clinically relevant oral formulations are warranted to assess translational feasibility. The identification of Mogat1 as a target of MAG opens new avenues for the development of MAG-based therapeutics or structural derivatives with enhanced potency and selectivity for NAFLD treatment.

We acknowledge that MAG, as a polyphenolic compound, possesses antioxidant properties that could potentially confound *in vitro* results through non-specific mechanisms such as pan-assay interference compounds (Baell and Walters, 2014). To mitigate this concern, we emphasize the following: (i) all *in vitro* findings were validated at multiple concentrations and were consistent with *in vivo* results, where systemic metabolism and pharmacokinetics reduce the likelihood of artifactual interference; (ii) the identification of MOGAT1 as a specific downstream effector was derived from unbiased RNA-seq analysis rather than a hypothesis-driven screening assay; and (iii) rescue experiments using MOGAT1 overexpression demonstrated that MAG’s effects are mediated through a defined molecular pathway, strongly arguing against non-specific antioxidant artifacts. Collectively, these lines of evidence support the specificity and biological relevance of our findings.

Several limitations of our study should be acknowledged. First, we acknowledge that our data demonstrate functional dependence rather than direct physical binding. We have not established a direct molecular interaction between MAG and MOGAT1 or its transcriptional regulators; future studies employing surface plasmon resonance, cellular thermal shift assays, or molecular docking are warranted to determine whether MAG directly binds to Mogat1 or modulates its transcription via upstream regulators. Second, while we focused on MOGAT1, RNA-seq data suggest that MAG may regulate other lipid metabolism genes; whether these contribute to the overall hepatoprotective effect warrants further investigation. Of note, the 7-day treatment regimen in our study primarily reflects suppression of ongoing fibrogenic activity rather than regression of established fibrosis, as the HFD model typically induces relatively mild fibrosis. Future studies employing alternative fibrosis models—such as CCl_4_-induced chronic liver injury or methionine-choline-deficient diet—with longer treatment durations will be necessary to determine whether MAG can reverse pre-existing fibrosis. Fourth, our *in vitro* experiments using AML12 hepatocytes primarily reflect hepatocyte-intrinsic changes in fibrogenic gene expression. Liver fibrosis is a multicellular process involving hepatic stellate cells, Kupffer cells, and extracellular matrix remodeling; future studies using co-culture systems or animal fibrosis models are needed to fully capture the anti-fibrogenic potential of MAG. Meanwhile, the correlations between MOGAT1 and lipid/inflammatory parameters were derived from pooled group data and may be influenced by between-group differences. Within-group analyses showed that MOGAT1 consistently correlated with TNF-α in both HFD and HFD + MAG groups, but not with TG or ALT, suggesting that the link between MOGAT1 and inflammation is more robust (data not shown). Importantly, the causal role of Mogat1 was demonstrated by rescue experiments, not by correlation analysis. Finally, the clinical relevance of our findings needs to be confirmed in human studies or using human hepatocyte models.

In conclusion, our study demonstrates that MAG alleviates HFD-induced hepatic steatosis, inflammation, and fibrosis by suppressing MOGAT1-mediated triglyceride synthesis and subsequent NF-κB-driven inflammatory responses. These findings identify MAG as a promising therapeutic agent and MOGAT1 as a novel effector for NAFLD intervention, providing a strong rationale for further preclinical and clinical development.

## Data Availability

The datasets presented in this article are not readily available because the raw RNA-seq data are part of an ongoing research project and are subject to institutional and intellectual property restrictions. Requests to access the datasets should be directed to the corresponding author Zhihua Xiao (xiaozhihua2026@163.com).

## Glossary

ACTA2: Actin alpha 2, smooth muscle
ALT: Alanine aminotransferase
ANOVA: Analysis of variance
AST: Aspartate aminotransferase
COL1: Collagen type I
DAPI: 4′,6-Diamidino-2-phenylindole
DHE: Dihydroethidium
DMEM: Dulbecco’s Modified Eagle’s Medium
FBS: Fetal bovine serum
H&E: Hematoxylin and eosin
HFD: High-fat diet
HPLC: High Performance Liquid Chromatography
HRP: Horseradish peroxidase
IL-1β: Interleukin-1β
IL-6: Interleukin-6
LDL: Low-density lipoprotein
MAG: Magnolol
MOGAT1: Monoacylglycerol O-acyltransferase 1
NAFLD: Non-alcoholic fatty liver disease
NASH: Non-alcoholic steatohepatitis
NF-κB: Nuclear factor kappa-B
PA: Palmitic acid
PBS: Phosphate-buffered saline
PCR: Polymerase chain reaction
PVDF: Polyvinylidene difluoride
RNA-seq: RNA sequencing
ROS: Reactive oxygen species
SD: Standard deviation
SDS: Sodium dodecyl sulfate
SPF: Specific pathogen-free
TBS-T: Tris-buffered saline with Tween 20
TCH: Total cholesterol
TG: Triglyceride
TGF-β1: Transforming growth factor-β1
TNF-α: Tumor necrosis factor-α
